# Effects of Water Addition on a Catalytic Fluorination of Dienamine

**DOI:** 10.3390/molecules24193428

**Published:** 2019-09-21

**Authors:** Daiki Kuraoku, Tsunaki Yonamine, Genta Koja, Norio Yoshida, Satoru Arimitsu, Masahiro Higashi

**Affiliations:** 1 Graduate School of Engineering and Science, University of the Ryukyus, Senbaru 1, Nishihara, Okinawa 903-0213, Japan; k198363@eve.u-ryukyu.ac.jp (D.K.); k188363@eve.u-ryukyu.ac.jp (T.Y.); 2 Center for Research Advancement and Collaboration, University of the Ryukyus, Senbaru 1, Nishihara, Okinawa 903-0213, Japan; kojagen@lab.u-ryukyu.ac.jp; 3 Department of Chemistry, Graduate School of Science, Kyushu University, 744 Motooka, Nishiku, Fukuoka 819-0395, Japan; noriwo@chem.kyushu-univ.jp; 4 Department of Molecular Engineering, Kyoto University, Kyoto daigaku-katsura, Nishikyo-ku, Kyoto 615-8510, Japan

**Keywords:** water addition, dienamine, fluorination, 3D-RISM-SCF, solvent effect

## Abstract

We investigate the effects of water addition on a highly stereocontrolled fluorination of dienamine generated by α-branched enals and 6′-hydroxy-9-amino-9-deoxy-*epi*-quinidine with *N*-fluorobenzenesulfonimide (NFSI) in the presence of Brønsted acid both experimentally and theoretically. It is experimentally found that water addition to organic solvent significantly shortens the reaction time whereas excessive water addition decreases the enantiomeric excess. The results calculated with three-dimensional reference interaction site model self-consistent field (3D-RISM-SCF) method are in good agreement with the experimental ones. It is revealed that the shortness of reaction time is caused by the reactant destabilization and that the decrease in enantiomeric excess is due to the difference of hydration free energy between two transition states.

## 1. Introduction

In organic synthesis, water addition into organic solvents often enhances the reactivity and the selectivity [1,2,3,4,5,6]. For example, in triple catalytic cross-coupling reaction developed by MacMillan group, addition of 40 equiv. of water into DMSO solvent increases product yield from 42% to 85% [3]. Maruoka et al. found that water addition for asymmetric aldol reactions with a chiral organocatalyst greatly increases the enantiomeric excess up to ~90% ee [4]. Although such effects of water addition on the reactivity and selectivity are widely known, the understanding of detailed mechanisms is still limited.

**Scheme 1:**
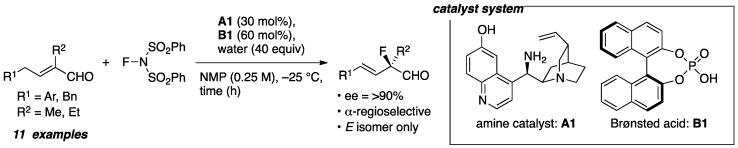
α-Fluorination of α-branched enals.

Recently, we developed the asymmetric fluorination of dienamines generated by α-branched enals and 6′-hydroxy-9-amino-9-deoxy-*epi*-quinidine in the presence of NFSI and Brønsted acid [7,8], and the reaction gave the excellent α-regioselectivity and enantioselectivity regardless of substituents on R^1^ and R^2^; thus, both electron-withdrawing and electron-donating groups on the aromatic ring of R^1^ have no significant influence, and all maintain excellent enantioselectivity over 90% ee. On the other hand, in the cases of benzyl group on R^1^ or ethyl on R^2^, the reactions gave slightly lower enantioselectivities, 81% and 77%, respectively (Scheme 1) [9]. During the optimization of the reaction condition, the addition of 40 equiv. of water was found effectively to shorten the reaction time maintaining the high stereoselectivity. In the previous density functional theory (DFT) investigation, we revealed the non-classical hydrogen bonding between C(sp^2^)–H and the counterion of the conjugate base of Brønsted acids is the key to stabilize the transition state for the major enantiomer, *R*-isomer determined by single-crystal X-ray diffraction analysis; however, the detailed role of water was remained unclear.

Therefore, in the present study, we investigate the deep insight of the effects of water addition on this fluorination reaction both experimentally and theoretically. First, changes in reaction time and enantiomeric excess by the amount of water are examined experimentally. Then the effects of water addition are analyzed by means of three-dimensional reference interaction site model self-consistent field (3D-RISM-SCF) method [10]. The 3D-RISM-SCF method is a combination of quantum chemical method and integral equation theory of liquids, which enables us to obtain complete ensemble average of solvation structure around a solute described quantum chemically. This type of approach has been successfully applied to chemical reactions not only in pure solvents but also in mixed solvents [11,12,13,14]. The free energy change due to the water addition is examined by decomposition analysis.

## 2. Results and Discussion

**Scheme 2:**
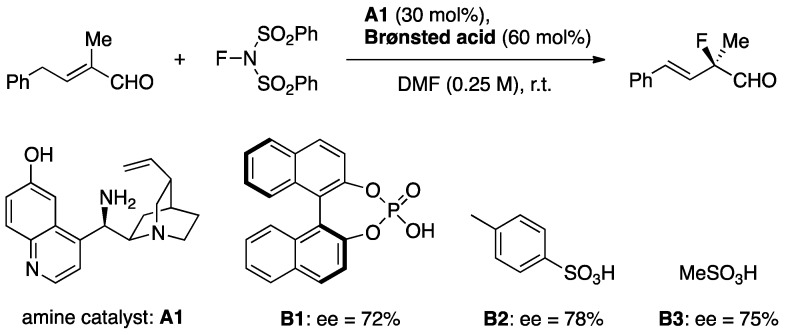
Effect of Brønsted acid on the enantioselectivity.

First, we investigated the effect of water on the fluorination reaction experimentally. We liked to run the reaction in DMF instead of NMP to obtain a comparable data of the later DFT calculation. Furthermore, we examined the effect of Brønsted acids for the enantioselectivity if we can find the simple acids give similar enantioselectivity so that we can reduce the computational time; thus, we examined three kinds of Brønsted acids, BINOL-based phosphoric acid (**B1**), *p*-TsOH (**B2**) and MsOH (**B3**) with amine catalyst (**A1**) in DMF as the solvent at room temperature without water (Scheme 2). Interestingly, the enantioselectivities of the corresponding product were obtained at 72% with **B1**, 78% with **B2** and 75% with **B3**, therefore Brønsted acids, **B2** and **B3** can be the good surrogates for **B1**.

**Figure 1:**
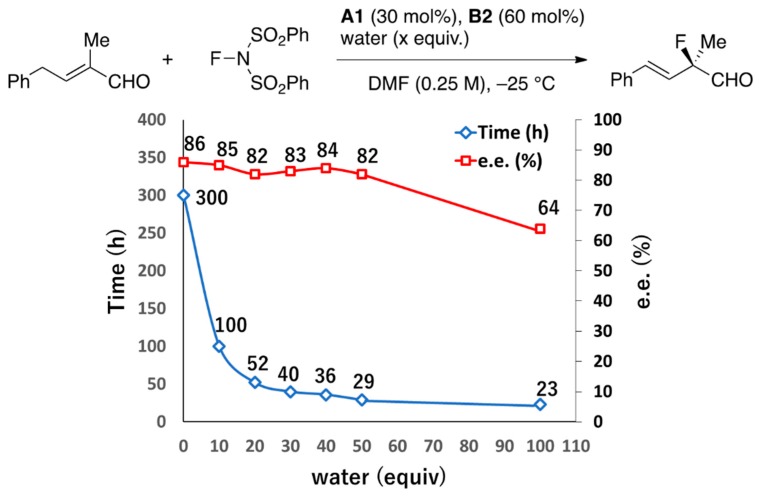
Experimental results of water addition.

Based on this control experiment, we examined the effect of water using **B2** as the Brønsted acid at −25 °C. The reaction without water did not consume the starting aldehyde even after 300 h; however, adding some water to DMF solution gave a shorter reaction time, as shown in Figure 1. On the other hand, the enantioselectivity outcomes were not changed until 40 equivalents of water was added, while a large excess of water addition decreases the enantiomeric excess drastically.

**Figure 2:**
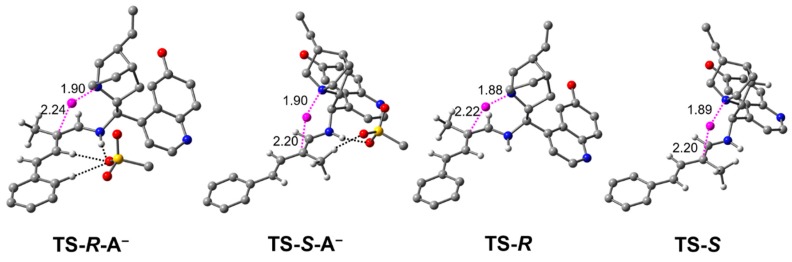
Calculated transition state (TS) geometries Breaking and forming bonds (purple, in Å) and important hydrogen bonds (black) are shown with dotted lines. Adapted with permission from [9]. Copyright 2017 American Chemical Society.

Next, we investigated the effects of water addition with the 3D-RISM-SCF method. Our previous study showed that two catalysts, quinuclidine and counterion of Brønsted acid, control the regio- and stereoselectivity at the transition state (TS) in a concerted manner (Figure 2) [9]. The fluorination proceeds at the α position because of the quinuclidine located close to the α position whereas the counterion preferentially stabilizes the **TS-*R*** configuration connecting to the major product due to the nonclassical CH hydrogen bonds. Following these results, we here considered four transition states, **TS-*R*-A^−^**, **TS-*S*-A^−^**, **TS-*R***, and **TS-*S***, in which “-**A^−^**” indicates the counterion is bound with the TS substrate.

**Figure 3:**
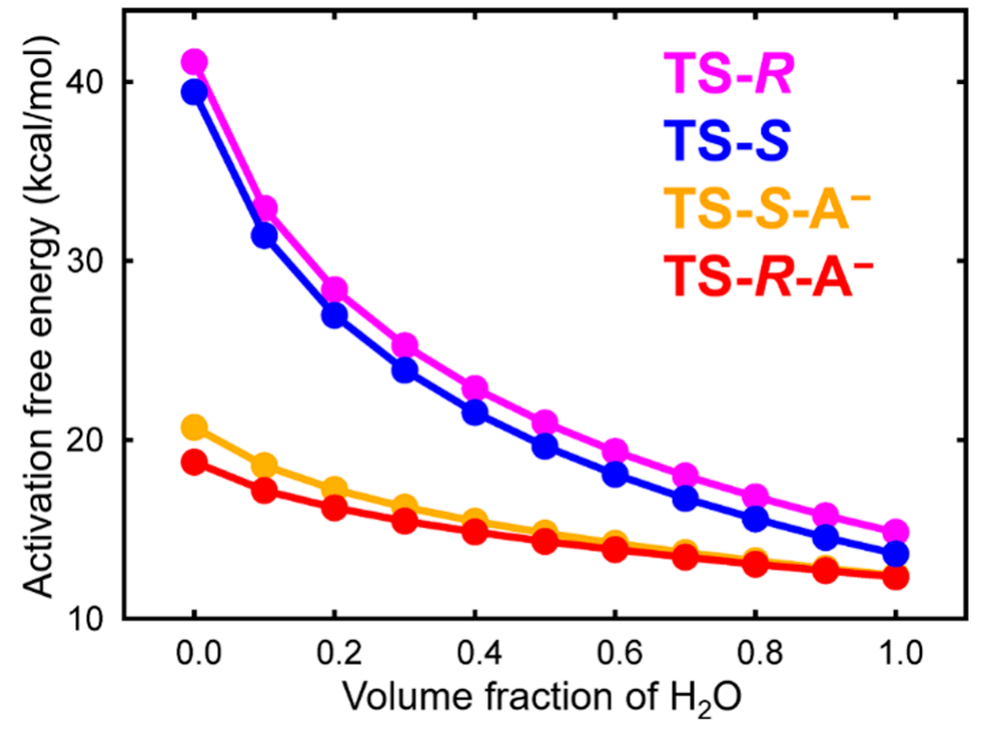
Calculated activation free energies with respect to volume fraction of H_2_O. The activation free energies of **TS-*R*-A^−^**, **TS-*S*-A^−^**, **TS-*R***, and **TS-*S*** are shown in red, orange, purple, and blue lines, respectively.

The calculated activation free energies with respect to volume fraction of water, x(H_2_O), are shown in Figure 3. In pure DMF solution, x(H_2_O) = 0, the activation free energies of **TS-*R*-A^−^**, **TS-*S*-A^−^**, **TS-*R***, and **TS-*S*** are 18.76, 20.70, 41.12, and 39.44 kcal/mol, respectively. The activation free energy of **TS-*R*-A^−^** is the lowest and ~2 kcal/mol lower than that **TS-*S*-A^−^**. In contrast, the activation free energy of **TS-*R*** is higher than that of **TS-*S***. This result indicates that the **TS-*R*** configuration is more stabilized with the counterion, which is consistent with our previous result [9]. It is noted that the counterion strongly interacts with the TS substrate in pure DMF solution. The free energy differences between with and without the counter anion are ~20 kcal/mol.

As the volume fraction of water is increased, the activation free energies of all the TSs is monotonically decreased. This result corresponds to the experimental result of shorter reaction time by water addition. It is also found that the free energy differences between the four TS states are decreased as the ratio of water is increased. In pure aqueous solution, x(H_2_O) = 1, the activation free energies of **TS-*R*-A^−^**, **TS-*S*-A^−^**, **TS-*R***, and **TS-*S*** are 12.35, 12.43, 14.84, and 13.63 kcal/mol, respectively. Although the **TS-*R*-A^−^** state has the lowest free energy in all the solutions, the free energy difference between **TS-*R*-A^−^** and **TS-*S*-A^−^** is gradually decreased and only ~0.1 kcal/mol in pure aqueous solution. Furthermore, the free energy differences between with and without the counter anion are also decreased, indicating that the interaction between the TS substrate and counterion becomes weaker. As a result, the activation free energies of the two TSs giving the minor product, **TS-*S*-A^−^** and **TS-*S***, approach to that of **TS-*R*-A^−^**. This result qualitatively agrees with the experimental result of decrease in enantiomeric excess by water addition.

**Figure 4:**
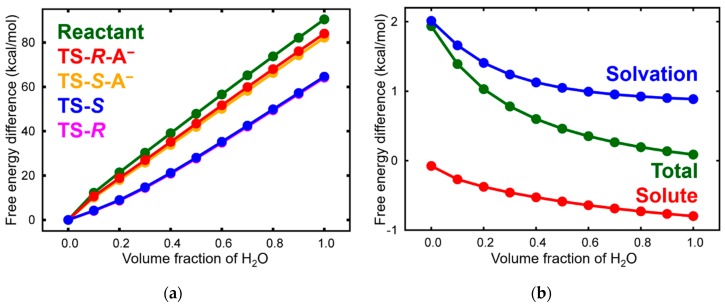
(**a**) Calculated free energy changes of reactant and TSs. The free energies at x(H_2_O) = 0 (pure DMF solution) are set to be zero. The free energy differences of reactant, **TS-*R*-A^−^**, **TS-*S*-A^−^**, **TS-*R***, and **TS-*S*** are shown in green, red, orange, purple, and blue lines, respectively. (**b**) Differences of activation free energy, solute internal energy and solvation free energy between **TS-*R*-A^−^** and **TS-*S*-A^−^**. Differences of activation free energy, solute internal energy and solvation free energy are shown in green, red, and blue lines, respectively.

To investigate the effects of water addition in more detail, we calculated the free energy changes of reactant and TSs with respect to the volume fraction of water (Figure 4a). It is found that both the reactant and TSs become unstable by water addition because they have many nonpolar parts. The lesser polar reactant is more destabilized than the TSs. Therefore, the decrease in activation free energy is caused by a large destabilization of the reactant. In addition, the TS complexes with counterion, **TS-*R*-A^−^** and **TS-*S*-A^−^** become more unstable than the TSs without counterion, **TS-*R*** and **TS-*S***, because the cationic **TS-*R*** and **TS-*S*** and anionic counterion can strongly interact with water solvent separately. As a result, the free energy differences between with and without the counter anion are decreased as the ratio of water is increased.

In the 3D-RISM-SCF theory, free energy of a solute is described as the sum of solute internal energy and solvation free energy. Figure 4b shows that how these two terms contribute to the decrease in free energy difference between **TS-*R*-A^−^** and **TS-*S*-A^−^**. When the volume fraction of water is small, the solvation free energy largely contributes to the free energy difference. As the ratio of water is increased, the positive solvation and negative solute contributions cancel each other, resulting in that the total free energy difference becomes nearly zero.

**Figure 5:**
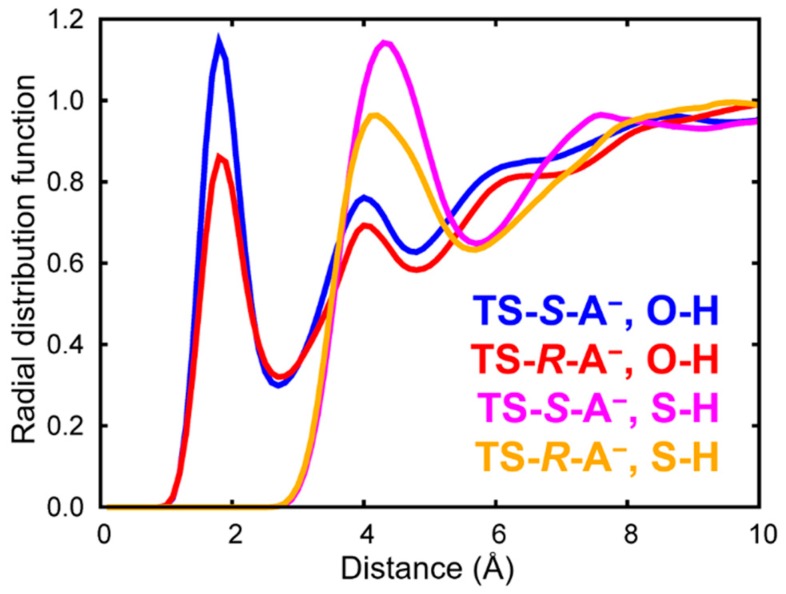
Calculated RDFs for the O or S (MeSO_3_^−^)-H (H_2_O) distances at x(H_2_O) = 0.3 volume fraction at **TS-*R*-A^−^** and **TS-*S*-A^−^**. The RDFs for the O-H distances at **TS-*R*-A^−^** and **TS-*S*-A^−^** and the S-H distances at **TS-*R*-A^−^** and **TS-*S*-A^−^** are shown in red, blue, orange, and purple, respectively.

We further analyzed the free energy differences between x(H_2_O) = 0.0 (pure DMF solution) and x(H_2_O) = 0.3, where the solvation contribution is dominant. The free energy difference between **TS-*R*-A^−^** and **TS-*S*-A^−^** is decreased from 2.10 kcal/mol at x(H_2_O) = 0.0 to 0.95 kcal/mol at x(H_2_O) = 0.3. Since the solvation free energy is approximated by a half of solute-solvent binding energy, we analyzed the difference of the binding energy between TSs and solvent, ΔE_bind_ = E_bind_(**TS-*S*-A^−^**) − E_bind_(**TS-*R*-A^−^**) in the two solutions. Table 1 summarizes ΔE_bind_ and its components. We found that the interactions between MeSO_3_^−^ counterion and solvent molecules particularly contribute to reducing the binding energy difference. Figure 5 shows the radial distribution functions (RDFs) for the S or O (MeSO_3_^−^)-H (H_2_O) distance at x(H_2_O) = 0.3. The first peaks at the **TS-*S*-A^−^** configuration are larger than those at the **TS-*R*-A^−^** configuration, indicating that the MeSO_3_^−^ counterion at the **TS-*S*-A^−^** configuration interacts with water molecules more strongly. It is noted that the MeSO_3_^−^ counterion at the **TS-*R*-A^−^** configuration preferentially stabilizes the TS-***R*** substrate and thus interacts with water solvent only weakly. Therefore, we concluded that the decrease in activation free energy difference between **TS-*R*-A^−^** and **TS-*S*-A^−^** by water addition is due to the difference of the interactions between MeSO_3_^−^ counterion and water molecules.

## 3. Materials and Methods

### 3.1. Experimental Methods

A suspension of **A1** (30 mol%) and **B2** (60 mol%) in DMF (0.3 mL, 3/4 volume of 0.25 M) was added to a controlled amount of water and stirred for 10 min at room temperature. NFSI (0.15 mmol, 1.5 equiv.) was added to the reaction mixture at 0 °C, and the whole solution was stirred for 15 min at 0 °C. The reaction mixture was cooled to −25 °C, and the solution of aldehyde **1** (0.1 mmol, 1.0 equiv.) in DMF (0.1 mL, 1/4 volume of 0.25 M) was added at −25 °C. The whole solution was stirred at −25 °C until the consumption of aldehyde was revealed by a thin-layer chromatography (TLC). To a reaction mixture was added Me_2_S at −25 °C, and then the whole reaction mixture stirred for 30 min at −25 °C. The resulting mixture was quenched with *sat*. NaHCO_3_ and extracted with Et_2_O. The combined organic phase was washed with brine and dried over MgSO_4_. The solution was filtered and concentrated with the rotary evaporator at 400 mbar, 35 °C. The residue was purified by a silica gel flash chromatography eluting with hexane/ethyl acetate = 7/1 to obtain the corresponding fluorinated aldehyde to determined yield of the product. Next, to a solution of the corresponding fluorinated aldehyde in DCM (1.0 mL, 0.1 M) were added AcOH (0.5 mmol, 5.0 equiv.) and benzylamine (0.5 mmol, 5.0 equiv.), and the whole solution was stirred for 1 h at the room temperature. Then, sodium triacetoxyborohydride (0.5 mmol, 5.0 equiv.) was added to the reaction mixture at the room temperature. The mixture was stirred under the argon atmosphere for 15 h at the room temperature. The reaction was quenched with *sat.* Na_2_CO_3_ and extracted with DCM. The combined organic phase was washed with brine and dried over MgSO_4_. The organic solution was filtered and concentrated under the reduced pressure. The residue was purified by a silica gel flash chromatography eluting with hexane/ethyl acetate = 7/1, then Enantiomeric purity was determined by a high performance liquid chromatography (HPLC) performed on JASCO PU-41810 and UV-4075 with Daicel Chiralpak IA-3, hexane/2-propanol/diethylamine = 100/0.3/0.1, flow rate = 1.0 mL/min, wavelength = 249 nm, retention time; 12.5 min (minor) and 14.6 min (major).

### 3.2. Computational Methods

We employed the 3D-RISM-SCF method [10] for the free energy calculations of solutes in the mixture of water and DMF. Notably, several previous studies showed that water molecules directly involve organic reactions [5,6]. However, we here assumed that the added water acts as a solvent because the enantiomeric excess is only gradually changed by water addition. It is noted that the hydrogen-bond interactions between substrate and water molecules are properly treated in the framework of 3D-RISM-SCF method. To reduce computational cost, MeSO_3_H (**B3**) was used as Brønsted acid. The density functional theory with M06-2X functional and 6-31G(d,p) basis set was employed for the electronic structure calculation of solutes. The solute geometries were taken from our previous study [9], in which the geometries were optimized at the SMD(DMF)-M06-2X/6-31G(d,p) level. The thermal corrections to the free energy were also added at the SMD(DMF)-M06-2X/6-31G (d,p) level. The Lennard-Jones parameters for solutes were taken from the general AMBER force field (GAFF) [15]. The simple point charge (SPC) model [16] with modified hydrogen parameters (σ = 1.0 Å and ε = 0.056 kcal/mol) and six-interaction site optimized potential computational model (CS2) [17] were used for water and DMF solvent, respectively. The volume fractions of water, x(H_2_O), were set from 0.0 (pure DMF) to 1.0 (pure water) at intervals of 0.1, where we assumed that the volumes of water and DMF in mixed solution are identical to those of pure solvents. The Kovalenko-Hirata closure [18] was used to solve the 3D-RISM equation. The temperature was set at 300 K. The grid points in the 3D-RISM-SCF calculations were 128 × 128 × 128 with a spacing of 0.5 Å. All the calculations were performed with a modified version of the GAMESS version 18 AUG 2016 (R1) program package [19], where the 3D-RISM-SCF method have been implemented [20].

## 4. Conclusions

In this article, we investigated the effects of water addition on the highly stereocontrolled fluorination both experimentally and theoretically. The experimental results are in good agreement with the calculated ones. It was revealed that the shortness of reaction time is caused by the reactant destabilization and that the decrease in enantiomeric excess is due to the difference of solute-solvent interaction between two transition states.

As noted in the Introduction, adding water into organic reaction systems often improves reactivity and selectivity. Notably, it is difficult to investigate such effects with a simple polarized continuum model, though it is widely used for the analysis of solvation effects. As the present study demonstrated, the 3D-RISM-SCF method is expected to be a powerful tool for studying the effects of water addition.

## Figures and Tables

**Table 1:** Analysis of the binding energy difference between **TS-*R*-A^−^** and **TS-*S*-A^−^** in the two solutions, x(H_2_O) = 0.0 and x(H_2_O) = 0.3 (in kcal/mol).

	ΔE_bind_(x(H_2_O) = 0.0)	ΔE_bind_(x(H_2_O) = 0.3)	ΔΔE_bind_^1^
aldehyde	−0.99	−0.82	−0.18
amine	1.49	3.37	−1.88
F atom	−1.22	−2.45	1.23
counterion	2.81	1.00	1.82
sum	2.09	1.10	0.99

^1^ ΔΔE_bind_ = ΔE_bind_(x(H_2_O) = 0.0) − ΔE_bind_(x(H_2_O) = 0.3).
